# Method of Using the Correlation between the Surface Roughness of Metallic Materials and the Sound Generated during the Controlled Machining Process

**DOI:** 10.3390/ma15030823

**Published:** 2022-01-21

**Authors:** Volodymyr Nahornyi, Anton Panda, Jan Valíček, Marta Harničárová, Milena Kušnerová, Iveta Pandová, Stanislaw Legutko, Zuzana Palková, Ondrej Lukáč

**Affiliations:** 1 Faculty of Electronics and Information Technologies, Department of Computer Science, Sumy State University, Rimsky-Korsakov, 2, 44007 Sumy, Ukraine; v.nahornyi@cs.sumdu.edu.ua; 2 Faculty of Manufacturing Technology with Seat in Prešov, Department of Automobile and Manufacture Technologies, Technical University of Košice, Štúrova 31, 080 01 Prešov, Slovakia; anton.panda@tuke.sk (A.P.); iveta.pandova@tuke.sk (I.P.); 3 Department of Mechanical Engineering, Faculty of Technology, Institute of Technology and Business in České Budějovice, Okružní 10, 370 01 České Budějovice, Czech Republic; jan.valicek@uniag.sk (J.V.); kusnerova.milena@mail.vstecb.cz (M.K.); zuzana.palkova@mail.vstecb.cz (Z.P.); 4 Institute of Electrical Engineering, Automation, Informatics and Physics, Faculty of Engineering, Slovak University of Agriculture in Nitra, Tr. A. Hlinku 2, 949 76 Nitra, Slovakia; ondrej.lukac@uniag.sk; 5 Faculty of Mechanical Engineering, Poznan University of Technology, 60-965 Poznan, Poland; stanislaw.legutko@put.poznan.pl

**Keywords:** steel, CNC lathe, generated sound, surface roughness, tool wear, adaptive control of the cutting process

## Abstract

The article aims to use the generated sound as operational information needed for adaptive control of the metalworking process and early monitoring and diagnosis of the condition of the machined materials using a newly introduced surface roughness quality index due to the sound-controlled machining process. The object of the measurement was correlation between the sound intensity generated during cutting and the material parameters of the machined surface, i.e., the roughness of the machined surface and the degree of wear of the cutting tool. The roughness was measured during longitudinal turning of a steel billet with a P25 insert made of 12X18H10T steel and a T15K6 cutting insert made of a titanium, cobalt, and tungsten group alloy. The correlation between the sound and roughness of the machined surface was 0.93, whereas between the sound and wear of the cutting tool was 0.93. The correlation between sound and tool wear in the experiment with P25 and T15K6 cutting inserts and the correlation between sound and roughness is positive.

## 1. Introduction

High demands are placed on machine tools not only in terms of required technological parameters and efficiency but also in terms of their reliability. The key to ensuring reliable production is timely and correct operational diagnostics and self-diagnostics of modern machine tools. Machine tools are currently the most demanding application in an unattended mode with continuous operation, in which the diagnostic system must quickly detect a dangerous trend of a monitored parameter or even a sudden failure. In principle, the diagnostic system communicates with the machine tool system at such a level that it is possible to start the prepared program sequence in time, minimising or averting the fault based on the current diagnostic results. An external diagnostic system can be used for the specific diagnostic needs of the machining system. The design of the additional system is the content of the presented publication.

### 1.1. Adaptive Control Methods

In particular, its implementation presupposes the connection of a computer with a diagnostic monitoring system, with measuring modules and signal-processing modules. Analysis of an acoustic signal can identify typical machine damage, such as imbalance, misalignment, bearing problems, gearing problems, loosening, electric motor problems, general drives, and more. The noise emitted by the machine provides partial information about its state. Vibration in machines can cause failure. With the help of vibrations and sound, it is possible to predict the condition of the surface and address problems that engineers and users have to solve in the process of making various technologies, especially when implementing control automation to maximise the performance during the production and determine the values of process parameters that are placed on the required quality of products [1,2]. Diagnostic and prediction methods for periodic measurement of vibration (noise) use vibration and noise as diagnostic parameters [3]. It is necessary to monitor the quality of machining products by cutting throughout the technological chain because it results from the complex action of many technological influences. The cutting process results in changing the machined surface and the surface of the machining tool interactively. In particular, the wear of the cutting tool has a major impact on the quality of the checked parameters. This fact requires the implementation of adaptive control to extend the warning time before the predicted failure of the machining operation.

The adaptive control system of the cutting process proceeds from the measurement of different information signals from nature representing a range of external factors: mechanical stress, vibration, the processing system elastic deformations, the electric current, chemical exposure, etcetera. These factors have a decisive impact on the degree of the tool wear and, consequently, on the manufactured product quality [4]. So, in the course of the tool wear process, such parameters as cutting force [5] and the torque [6,7] are changing. In order to measure these parameters, dynamometers are used. For example, when applying the vibration sensor when turning on the tool holder, the measurement is performed for tool vibrations inevitably accompanying the cutting process [8]. The interaction of the wearing tool with a workpiece leads to generating acoustic emission waves; acoustic emission sensors record them. Elastic waves are more sensitive to tool wear than force factors and vibration. However, at the same time, it results in making acoustic emission more sensitive to noise interference caused by the environmental impact and the work of the operational machine units. For this reason, the “integrated settings” are referred to as they are more resilient to noise disturbance. For example, thermocouples are used to monitoring the cutting zone temperature. The drawback of the “integral” methods is that they consist of large inertia and the need to incorporate a thermocouple and electrical connections into the instrument. In adaptive control, acoustic emission signals and measurements of vibrations are used as well [9,10]. However, in practice, using the signals is connected with a major problem that is difficult to solve. In order to record these information signals, the contact method is applied, which requires mechanical communication between the sensor and the information signal source (the object surface). The only exception is non-contact sensing in the case of magnetic sensors or sensors mounted by magnetic holders [11,12,13].

It is necessary to find an informative point; in the case of cutting, it is the point of contact between the tool and the workpiece. It is not possible to place the sensor precisely at this point, so other possibilities are sought for the nearest control points (e.g., on the cutting tool holder) that would exclude the most significant interferences from the measurement results. In addition, noise interference, which is always associated with the measurement method used, i.e., vibrations generated by the machine tool operating nodes, must be avoided. As this is a non-contact measurement method, the contact of the sensor with the vibrating surface of the machine is eliminated. The research issues have included the investigation of acoustic signals in tool steel-hardening [14] and cold-drawn steel [15], cutting tool stability against impacts [16], tool wear and surface roughness in milling [17], tool wear in drilling by acoustic emission [18], detection of contact mechanism changes due to grinding tool wear by acoustic emission [19], and detection of suitable cutting operations [20]. The close relationship between the parasitic machine tool vibrations and the machined surface roughness is clearly shown in [21]. The machined surface is never ideally smooth; its roughness is carefully examined. In practice, the surface roughness is declared an important parameter, especially for dynamically stressed components, which usually start to fail at the surface. Therefore, a higher roughness has an adverse effect on the fatigue strength of the components and possibly also on their resistance to abrasion. The need for non-contact control of the cutting process is illustrated by examples of the ineffectiveness of cutting processing control by other methods. In this regard, [22], proposed an additional vibrator be used, which dampens parasitic cutting tool vibrations at the point of contact with the workpiece surface. It significantly complicates the process of part manufacturing, which is unacceptable for mass production. In [23], the authors considered an even more complex system for suppressing parasitic vibrations by directly controlling the cutting force, which is also not acceptable in the practice of actual production.

The acoustic emission considered in [24] is very sensitive to the parasitic signals generated by a running machine and, therefore, is not acceptable for the cutting process adaptive control. In the paper [25], it was noted that the complexity of adaptive control depends on the uncertainty of tool wear and fluctuations in the material properties of the part, which cannot be resolved at present. Papers [22,26], proposed a vibrator be used to dampen parasitic vibrations, which is very difficult for implementation in industrial conditions. In the paper [27], a digital control system for the longitudinal spindle stiffness was considered, designed to dampen its parasitic vibrations, but it significantly complicates the machining process. The authors in the paper [28] proposed a sliding mode of adaptive control, demonstrating the complexity of adaptive control of the cutting process. The article [29] proposed counteracting parasitic vibrations using two piezo vibrators programmatically controlled by a neural system, which indicates the complexity of the observed cutting process. In the paper [30], an example of a neural network was considered, which makes it possible to calculate the cutting force based on the cutting conditions, and based on this, in turn, to estimate the tool service life and the machined surface roughness. The Wavelet-Extreme-Machine software package was proposed in [31], which allows controlling the cutting process on the basis of the cutting force measurement results. A complex computerised system of adaptive control was considered in the paper [32], which allows using a computer cloud to store information that makes it possible to control the processed surface quality, which is the best opportunity for material processing cutting. As shown in the papers [33,34], the quality of adaptive control depends on the successful control of cutting conditions, which is, in turn, ensured by the automation of the control process itself. The presently used relationships between the information signal and tool wear are phenomenological, frequently without quantitative substantiation. The research issues have been investigated in the context of machine learning for predictive maintenance in milling [35], development of a new algorithm for online tool wear control [36], development of an innovative, intelligent machine tool [37], adaptive speed control for waterjet milling [38], and model-free adaptive predictive control for CNC [39]. Therefore, at the present level of the development of material processing by cutting, it is necessary to determine the degree of correlation between the sound generated during the cutting process, i.e., one of the promising information signals, the wear of the cutter, and the workpiece surface roughness. The research issues have been mentioned in learning approaches to minimising milling cycle time [40], production process control to support workpiece surface quality during drilling [41], adaptronic applications in cutting machines [42], adaptive control of metalworking technology system operation [43], and dynamic-state control of metalworking technology systems [44,45].

### 1.2. Advanced Adaptive Control Methods

Modern adaptive control systems are particularly suitable in the case of insufficient data on the continuous variation in the technical status of the processing system and represent the possibility of online control, i.e., without interrupting the processing process. They are based on an indirect method of controlling the processing process, i.e., they use information signals produced by many different physical phenomena that organically accompany the processing process. The research issues were addressed in the framework of sensor signal methods for the monitoring of tool wear in predicting progressive tool wear and cutting tool breakage using acoustic emission and cutting force signals in turning [46], experimental study, and analysis of metal matrix composites’ machinability characteristics in drilling [47], optimisation of cutting forces in turning composite materials [48], and drive motor performance [49,50]. The research issues have been studied in a review of monitoring methods in the milling processes regarding tool condition [49], and machine learning for flatness deviation in automatic prediction for face mill tooth wear [50], as well as acoustic emission signals [51] and vibration [52]. Another study was dealing with the secondary hardening effect on cutting forces, cutting temperature, and tool wear during high-alloy tool steel hard turning [53]. Research issues have been investigated in publications focusing on the impact of oil mist feed on the cutting point temperature and tool wear in controlled rotary cutting [54], prediction of flank wear of milling tools by determining the temperature-dependent wear mechanism [55], the effect of nanofluid addition on tool life, tool wear, roughness, and turning temperature [56], materials, processes, and systems in modern manufacturing [57]. Furthermore, the infrared radiation of shavings [58] is directly and continuously monitored during cutting. The above methods of monitoring the machining process have a significant predictive value, which can be finally reflected in a reduced form in the monitoring of the ongoing condition of the cutting tool itself. However, the main task of the adaptive control of the dynamic behaviour of the machining system is to ensure the quality of the machined part desired by the drawing. Furthermore, one of the most important parameters that determine the quality of a part is the quality of its surface, quantitatively expressed by roughness parameters [59]. If the surface roughness values determined by product quality indicators [60,61,62] exceed the permissible limits, they are classified as a technological failure of the processing system. Research issues have been focused on the investigation of surface roughness values of turned steels [60], the use of spindle noise designed to monitor tool wear in the turning process [61], and the monitoring of tool flank wear during turning by SVD analysis on emitted sound signal [62]. The processing process monitoring methods in question are mainly applicable in laboratory conditions intended for research purposes because the sensor sets of the measuring and control system cause disturbances within the cutting process. The level of disturbance depends, for example, on probe placement, tool stiffness, materials, and operating modes. In addition to the specific operational problems, indirect control methods have the significant disadvantage of a lack of published “vibration activity standards”, especially in the case of methods developed for rotating machines. This circumstance has necessitated monitoring and using information signals generated by concurrent physical processes. An experienced machine operator is able to determine when optimum conditions cease to exist in the cut by simply listening [63] For example, it is not the noise level of the sound samples that is evaluated but their frequency characteristic, which is obtained using the Fourier transform. However, it is necessary first to consider which outputs are required and choose the length of the intervals to be examined accordingly [64]. However, investigating the cutting sound is one thing; the requirement to control the cutting process is another. In order to control the cutting process, the monitoring of trends in the temporal development of cutting sound intensity in correlation with trends in the temporal development of surface roughness and wear rate is newly used in the publication. Various modern monitoring systems are used to monitor the measured characteristics of the machining process and, at the same time, allow data to be stored, evaluated, and compared. Subsequently, it is possible to control the machining process by modifying the cutting conditions (by changing the cutting speed, feed rate, or coolant supply) and terminating the ongoing operation and preventing damage. The same role is played by non-contact measurement using a microphone connected to an Android tablet.

The research presented here mainly aims to perform the indirect and continuous assessment of the technical condition of the machined, inspected component, namely in a reliable, accurate, and disturbance-resistant manner. The presented results demonstrate that the sound waves generated during the cutting of materials meet these demanding requirements.

A standard CNC machine tool program is basically a set of information compiled in a particular sequence, which is used to control the entire cutting process of the desired part. Although this production process is very precise, by implementing information about the actual interaction between the material and the tool, a more efficient procedure for controlling the machining process is achieved. Primarily, the article aims to quantitatively describe the correlation between the sound produced during turning, tool wear, and the roughness of the machined surface. The novelty of the research lies in the determination of the coincidence between the trends of the sound waveform and the trends of the surface roughness profile and tool wear. The practical benefit of the research lies in the robustness of the sound to multiplicative, additive, and impulsive interferences and the almost complete elimination of rejects due to the early identification of the condition of the machined material (using the proposed machining quality index).

## 2. Theoretical Part

### 2.1. Process Diagram

**Figure 1:**
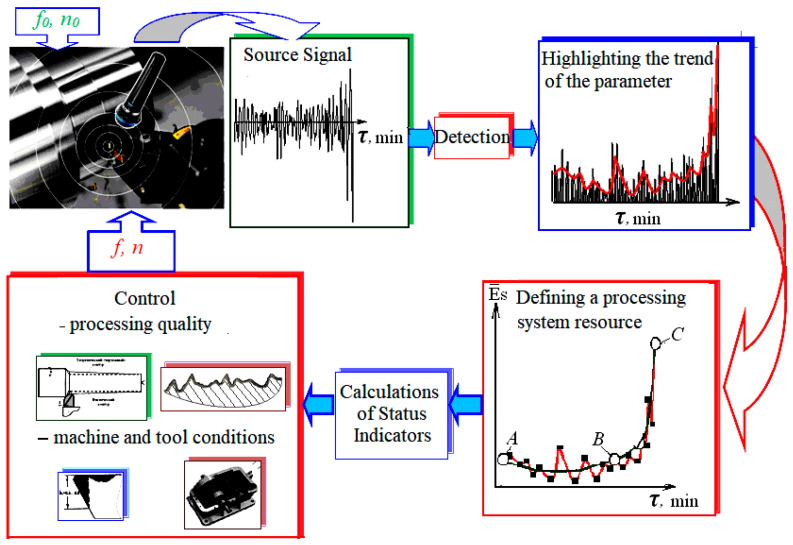
Process diagram, where *f*—adjustable feed rate, *n*—adjustable cutting speed, *f*_0_—initial feed rate and *n*_0_—initial cutting speed, ${\bar{E}}_{S}$—relative sound signal.

The process diagram (Figure 1) schematically depicts a comprehensive view of how sound and surface parameters are theoretically identified as source signals, then measured in practice and evaluated for correlation and finally used for feedback quality control of the technological process through feedback [50,51,52].

### 2.2. Sound Robustness to Noise Interference

The sound generated during cutting is recorded by the microphone, i.e., the voltage of the electrical signal at the microphone output changes depending on the pressure of the measured sound. The most suitable type of microphone is an electret microphone, in principle, an electrostatic microphone-based capacitor, in which the electric field is generated by a non-conductive mass permanently electrically charged, i.e., by an electret. The simple design of this microphone allows for convenient miniaturisation of its dimensions, while sensitivity at an acoustic frequency of 1 kHz electric voltage 1–10 mV to a sound pressure of 1 Pa is acceptable for the measurement.

There is a need to avoid the multiplicative noise interference; therefore, the measured microphone sound signal *E_S_* in a dimensionless form is used. This parameter is introduced by (1).

(1) $${\bar{E}}_{S}=\frac{E_{Si}}{E_{S0}^{}}$$

The extended relation (2) allows the elimination of the multiplicative noise fraction (1):

(2) $${\bar{E}}_{S}=\frac{E_{Si}\cdot \varepsilon \left(\tau \right)}{E_{S0}^{}\cdot \varepsilon \left(\tau \right)}=\frac{E_{Si}}{E_{S0}^{}}$$

*ε*(*τ*) is the corresponding duration of multiplicative noise.

Taking into the account the additive noise fraction, we obtained the form (3):

(3) $${\bar{E}}_{S}=\frac{\sqrt{{\hat{E}}_{Si}^{2}+{\hat{\varepsilon }}^{2}\left(\tau \right)}}{\sqrt{{\hat{E}}_{S0}^{2}+{\hat{\varepsilon }}^{2}\left(\tau \right)}}=\frac{{\hat{E}}_{Si}}{{\hat{E}}_{S0}}\frac{\sqrt{1+\frac{{\hat{\varepsilon }}^{2}\left(\tau \right)}{{\hat{E}}_{Si}^{2}}}}{\sqrt{1+\frac{{\hat{\varepsilon }}^{2}\left(\tau \right)}{{\hat{E}}_{S0}^{2}}}}$$

where the values ${\hat{E}}_{Si}$,  ε(τ)—or their respective RMS (root mean square) values—are the values of the useful, effective signal and noise (4)

(4) $${\hat{E}}_{S}=\frac{E_{S}}{\sqrt{2}};\;\hat{\varepsilon }{\left(\tau \right)}_{S}=\frac{\varepsilon \left(\tau \right)}{\sqrt{2}}$$

It is possible to meet strict requirements for accuracy of fit by the use of clamping screws with a head that has a negative shape of the cutting insert hole. Tapered shapes enable partial concentricity of fit. Such a solution is expensive because each type of cutting insert must have a specially designed clamping screw. When selecting the turning inserts, the following parameters were carefully considered: the geometry of the insert (depending on the operation to be performed); the largest possible angle of the insert tip (with respect to strength and economy); the size of the insert (depending on the depth of cut); the radius of curvature of the insert tip (with respect to the strength of the material and a smaller radius in case of tendencies to vibration). The impact of the correct choice of insert parameters on the final machining process is considerable, as good control of chip formation and machining performance is achieved.

In the considered case, the distance to the cutting zone of this machine from neighbouring machines (sources of interference) was 4 m. The total effective value of the sound pressure ${\hat{E}}_{S}^{SUM}$ (measured in pascals) at the checkpoint is determined analytically by the vector sum according to (5). The “with canopy” notation is the root-mean-square value of the useful signal and the interference. Thus, in this case, the root-mean-square value of the sound pressure at the control point is calculated using the following expression:

(5) $${\hat{E}}_{S}^{SUM}=\sqrt{{\hat{E}}_{S}^{2}+{\left(\frac{{\hat{E}}_{S}}{4}\right)}^{2}+{\left(\frac{{\hat{E}}_{S}}{4}\right)}^{2}}={\hat{E}}_{S}\sqrt{\frac{18}{16}}=1.06{\hat{E}}_{S}.$$

In the calculation of relation (5), the assumption was that in the process of work, three systems generated the sound of the same amplitude *Es*, Pa. Noise interference from the nearby devices leads to overestimating the results of measurements concerning the actual controlled sound magnitude value only by 6%.

According to (5), in the calculation, it is assumed that three process systems for sound pressure processing of the same amplitude *E_S_* (measured in Pa) were generated during the cutting process. It can be observed that noise disturbance due to the environment leads to overestimating the measurement results (for the actual value of the regulated acoustic value) by a maximum of 6%.

A total of 3072 signal values are determined in the sound management process to improve measurement reliability and noise interference control (reading is performed three times for a total of 1024 samples). Read values are summed and averaged to 3072 units. In order to determine the unit of the useful signal and the amount of noise, the amplitude values of the pressures at their effective value can be assumed to determine the overall useful signal level and interference (6):

(6) $${\hat{E}}_{S}^{SUM}=\sqrt{{\left({\hat{E}}_{S}\cdot \frac{2972}{3072}\right)}^{2}+{\left(10\cdot {\hat{E}}_{S}\cdot \frac{100}{3072}\right)}^{2}}=1.02{\hat{E}}_{S}.$$

It can be clearly seen that even if the noise level is exceeded ten times above the useful signal level, its contribution to the total signal is not higher than 2%. Thus, the studies mentioned above have shown that the noise generated during cutting has noise resistance properties and can, therefore, be considered a useful initial information signal for adaptive control of the cutting process. The cutting sound should provide a solution to the fundamental problem of adaptive control; maintaining the quality required for manufacturing parts in the documents, the desired degree of alignment of the part geometry, and the surface cleanliness are in accordance with the drawings. The geometric accuracy of the desired workpiece shape depends on tool wear and surface finish.

### 2.3. Control—Processing Quality

**Figure 2:**
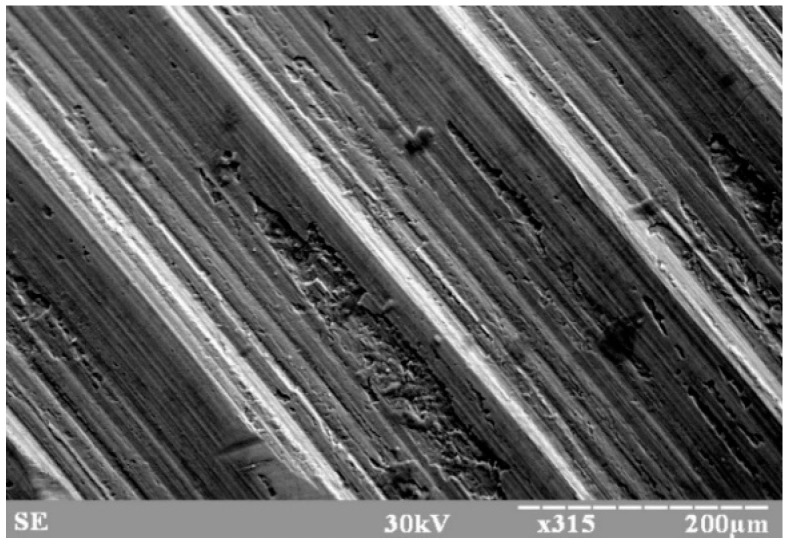
The surface of part of the workpiece after the cutting process and magnified by a microscope.

On the surface of the examined part of the workpiece, increased roughness was observed in the form of formed protrusions and depressions (Figure 2). Tool traces left on the machined surface were caused interactively, i.e., by tool vibration and workpiece vibration. This phenomenon was first recorded 135 years ago as a phonogram, recording the effect of the milling cutter on the lateral surface of the wax roll. Figure 2 was created with the REM-100U scanning electron microscope.

The altered surface roughness, similar to the phonogram acoustic recording, is inextricably linked to the destructive wear of the tool; therefore, it contains necessary information about the extent of this wear and the quality of the processed workpiece surface. The study of the possibility of using the acoustic signal in machining as an information signal about this process relates mainly to determining the noise resistance of the acoustic signal. It belongs among the main requirements for this kind of signal in adaptive control of cutting machining.

### 2.4. The Indicator of the Accuracy of Measurement Results Processing

One of the main reasons for deviation in the resulting geometrical shape of a part of the workpiece from its desired geometrical shape is the change in the cutting tooltip curvature radius *h_r_* corresponding to the machined surface curvature radius.

The presented experiments aimed to determine the possible correlation between the generated sound and tooltip wear during the machining of the workpiece on the lathe. The sound measurements were made using a microphone placed near the cutting area with signal transmission to the computer; the acoustic signal was measured continuously. Simultaneously, the value of the wear by canting the main rear surface of the tool (measured in mm) was recorded in two tool passes using a digital microscope for measurements. The experiment was stopped at the maximum permissible wear value. Thus, the correlation between the generated sound and the subject parameters was investigated experimentally.

### 2.5. The Indicator of the Accuracy of Measurement Results Processing

The method of similarity and dimension was used to obtain the analytical term “member function” describing the quality indicator for adaptive control *a_qp_*.

According to this theory, the desired indicator can be expressed using a set of determining parameters: initial wear $h_{r}(\tau_{0})$, current wear $h_{r}(\tau )$, current tool life τ, the numerical value of tool life ${\bar{T}}_{exp}$.

These parameters have two dimensions—length *L* ($\left[h_{r}(\tau_{0})\right]=L,\left[h_{r}(\tau )\right]=L$) and time *T* ($\left[\tau \right]=T,\left[{\bar{T}}_{exp}\right]=T$). The required exponent *a_qp_* can be presented as a function of the product of these determining parameters; each of these parameters is increased to its own degree (7):

(7) $$a_{qp}=f(h_{r}(\tau_{0}),h_{r}(\tau ),\tau ,{\bar{T}}_{exp})=h_{r}{}^{\alpha }(\tau_{0})\cdot h_{r}{}^{\beta }(\tau )\cdot \tau^{\gamma }\cdot {\bar{T}}_{exp}^{\lambda }.$$

Determining the rate indicator values is also necessary, i.e., replacing the quantities in the expression by their dimensions according to (8):

(8) $$1=L^{\alpha }\cdot L^{\beta }\cdot T^{\gamma }\cdot T^{\lambda }=L^{\alpha +\beta }\cdot T^{\gamma +\lambda }.$$

Because the desired indicator is a dimensionless quantity, its dimension, according to the dimension theory, is equal to one, whereas for exponents in the relation (9), the following conditions (9) must be met:

(9) α+β=0,γ+λ=0.

The above-mentioned set of two equations is further supplemented by four unknown conditions β=1,γ=1 whereby the common solution of fours equations (α=−1,β=1,γ=1,λ=−1) leads to the relation for the precision index, introduced here as the machining quality index *a_qp_* (10):

(10) $$a_{qp}=\frac{h_{r}(\tau )}{h_{r}(\tau_{0})}\cdot \frac{\tau }{{\bar{T}}_{exp}}.$$

According to (1)–(2) definition relations for a close correlation between the tool wear curve and the generated sound trend, the relation (9) can be reformulated to the form (11):

(11) $$a_{qp}=\frac{E_{Si}(\tau )}{E_{S0}(\tau_{0})}\cdot \frac{\tau }{{\bar{T}}_{exp}}={\bar{E}}_{S}(\tau )\cdot \frac{\tau }{{\bar{T}}_{exp}}.$$

The relation (10) is then a sought-after “member function” that varies according to the theory of “fuzzy sets” from zero to one [30]. The degree of change in the geometry of a workpiece component due to tooltip wear can be estimated by the relation (12):

(12) $$\delta =\frac{D_{\phi }-D_{H}}{D_{H}^{+\Delta B}-D_{H}}$$

where *D_H_* is the nominal size of the workpiece component; *D_ϕ_* is the actual size of the component ($D_{\phi }=D_{H}+2\cdot h_{r}$); *h_r_* is the radial wear of the tooltip; and Δ*B* is the change in radial wear of the tool. The relation (14) can be rewritten into the relation (13):

(13) $$\delta =\frac{D_{H}+2\cdot h_{r}-D_{H}}{D_{H}^{}+\Delta B-D_{H}}=\frac{2\cdot h_{r}}{\Delta B}.$$

The expression (13) varies from the 0 value when the tool is fully sharpened to 1 when the radial wear of the tool *h_r_* reaches the maximum allowable value. Thus, the machining quality index *a_qp_* (11) varying from zero to one unit adequately describes the degree of change in the geometry of the part and, for the first time in cutting practice, directly characterises the quality of adaptive control.

## 3. Experimental Part

**Figure 3:**
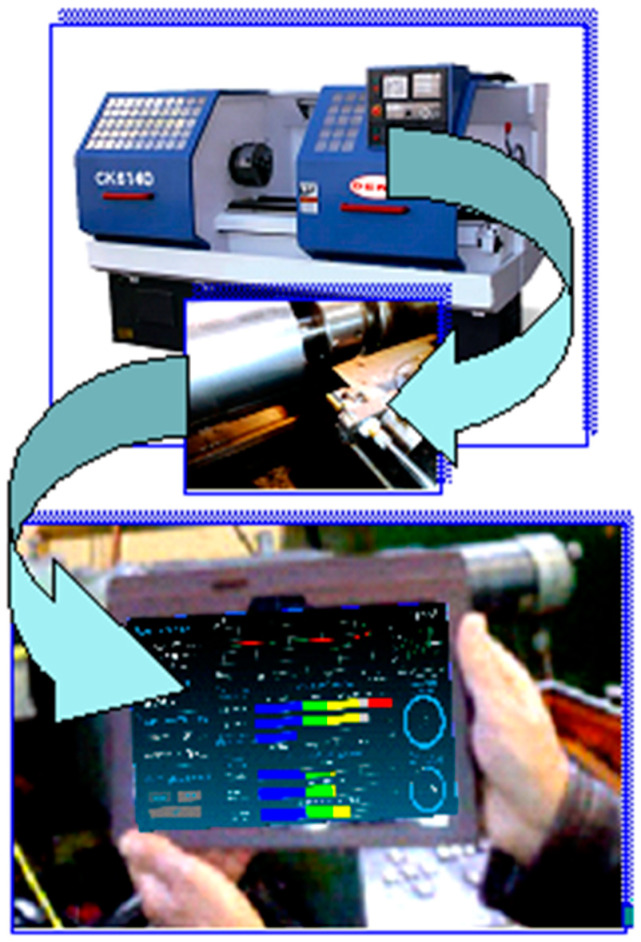
Automated monitoring of the technical conditions of the lathe.

The efficiency of adaptive control of the intensity of the generated sound by cutting was experimentally tested on a CNC lathe 16 K20T1 (Moscow machine-tool constructing factory, Moscow, Russia) (Figure 3). The adaptive control algorithm is shown in Figure 1. According to the algorithm, adaptive control begins by continuously registering the audio signal, selecting its envelope, and approximating with this envelope prediction model, which determines the source of the cutting tool as one of the parameters of the model. On the basis of the results of the operative comparison of the actual tool life and the required workpiece processing time, which ensures the quality required by the drawing, the initially selected cutting conditions were set.

The material and technical equipment were selected with the requirement of good availability, compatibility, and accuracy, with potential for future practical application. The following tools were selected: a CNC lathe 16 K20T1 machine tool with operating systems, an electret microphone, and a tablet with an Android operating system. The demonstrated material and technical equipment selection is only recommended as suitable for practice. The proposed method is of general validity and can be re-implemented by analogy with different combinations of the above material and technical security elements.

In accordance with the adaptive control algorithm, the sound level that is generated by the process of cutting was monitored in the experiment (Figure 3), and based on this, the value of the indicator *a_qp_* was calculated. Based on the value of this indicator, the quality of the adaptive section control was assessed. The experimental conditions are shown in Table 1. For all types of lathes, the basic cutting conditions for setting up the machining process are feed, depth of cut, and cutting speed.

Verification measurements were performed continuously for four years, specifically in each mode with ten repetitions, for a total of 280 measurements with an accuracy of 1 to 5%. From these measurements, two representative sets were created, the mean values of which were created by the graphical dependencies. In principle, the existence of a correlation between the intensity of the cutting sound and the roughness or wear rate is not dependent on the setting of the cutting conditions; the correlation always exists.

### 3.1. Sound Measurement

**Figure 4:**
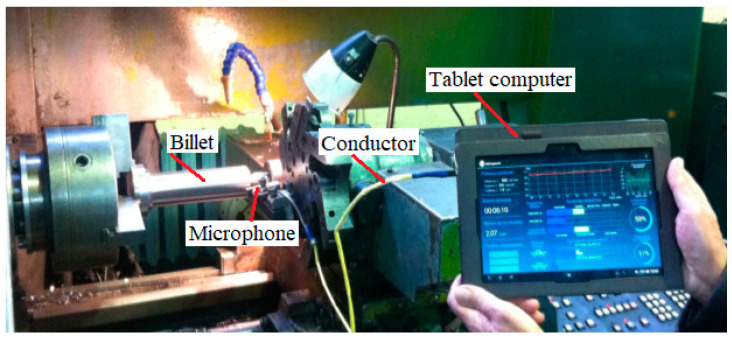
Sound control with a tablet.

The sound parameter *E_S_* was recorded using a program on the tablet (Lenovo Group Limited, Beijing, China) (Figure 4). The program implementing the derived algorithm is shown in Figure 1. The program is designed for the Android operating system. The first measurement of the generated sound (Figure 4) was made at the beginning of the cutting process (theoretically at *t* = 0 s).

**Figure 5:**
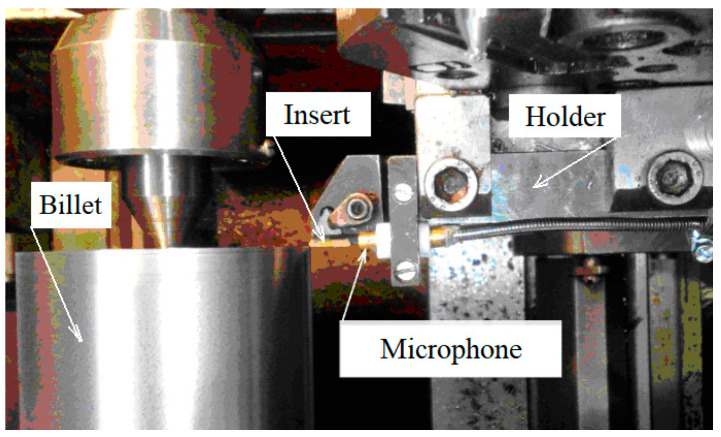
Installing the microphone in the immediate vicinity of the cut area.

During the experiment, the microphone was installed on the cutter holder (Figure 5) at a distance of 10 mm from the cutting zone.

The sound was measured by a microphone installed 1 cm from the cut area. The microphone is part of a newly developed cutting process control system because the generated sound is the only information signal that does not respond to the noise surrounding the cut area. In practice, it is realistic to have a large number of machine tools generating different sounds in one room. Therefore, a significant advantage of this method is that the results of acoustic measurements of the mixing of different sounds do not affect the applicability of the methodology.

### 3.2. Measurement of Surface Roughness

The presented experiment aimed to determine the correlation relationship between the dimensionless sound parameter *E_S_* and the height parameter *Ra* characterising roughness. The roughness was measured during longitudinal turning of the steel billet with a P25 insert and the cutting insert T15K6 made of titanium cobalt alloy and tungsten group (79% WC, 15% TiC and 6% Co). The workpiece material was made of 12X18H10T steel that includes manganese (0.64 in weight—%), silicon (0.39 in weight—%), titanium (0.42 in weight—%), chromium (17.8 in weight—%), nickel (9.45 in weight—%), and other trace elements such as phosphorus, sulphur, carbon, wolfram, and vanadium. The workpiece material’s hardness was 179 HRB, and the cutting inserts had a hardness of 419 HRB.

Two standardized (ISO 9361-1) interchangeable inserts were used to perform the turning experiments:

– Replaceable cutting insert T15K6 with a height of 10 mm and insert tip radius of 1 mm (type T—triangular);
– Replaceable cutting insert P25 with a height of 10 mm and insert tip radius of 1 mm (type P—pentagonal).

Measuring *Ra* parameters was performed periodically every five passes using a cutting profile using a profilometer 283. The measurement results were recorded using a dial indicator and recorded in a notebook. The signal recorded in the notebook was further processed to determine the correlation between:

- Sound trends and roughness parameters;
- The frequency spectrum of the roughness profile and sound;
- The temporal unfolding of the audio signal and roughness profile.

## 4. Results

### 4.1. Experiment with the Cutting Insert P25

**Figure 6:**
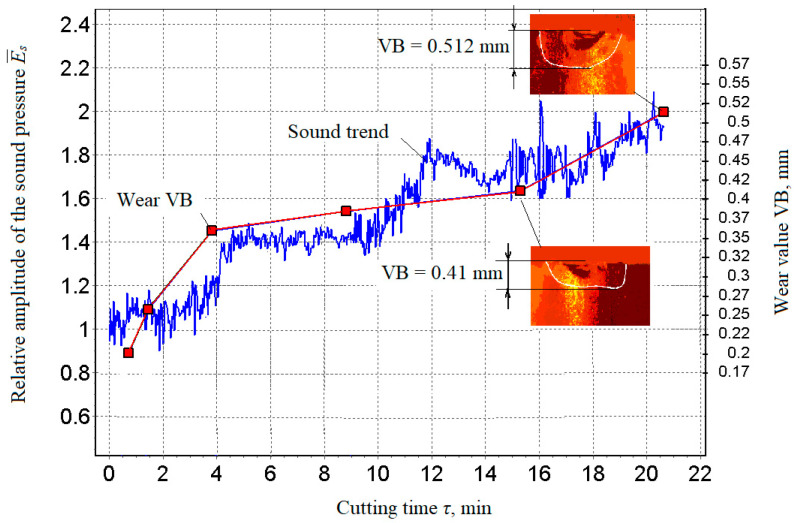
Trend of parameters and curve corresponding to the degree of wear VB.

The relationship between the tool wear curve and the development trend of the generated sound during cutting was determined using a P25 insert/plate for the following modes: (330 m∙min^−1^, 0.15 mm∙rev^−1^, 1.0 mm (Figure 6)); i.e., a sample diagram of the cutting sound change (the trend of parameters) and the wear curve VB.

**Figure 7:**
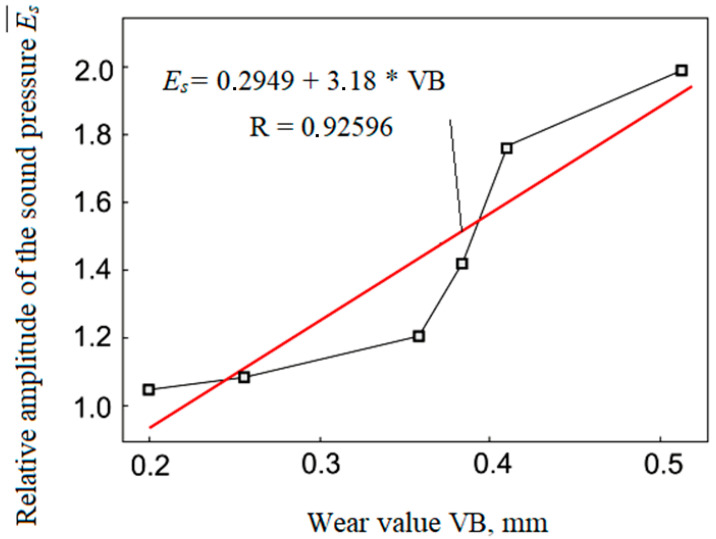
Correlation dependence between parameter ${\bar{E}}_{3B}$ and size of flank wear VB.

**Figure 8:**
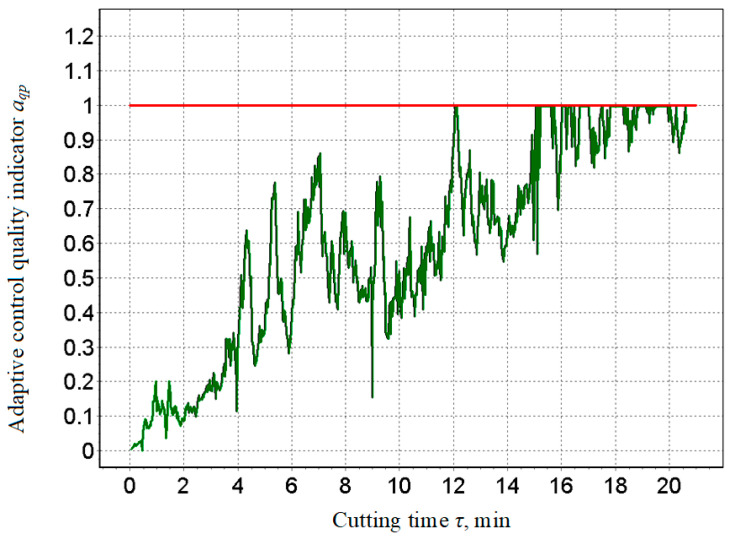
A quality indicator for adaptive control *a_qp_*.

From the dependence (Figure 7), it was possible to identify a clear relationship between sound and wear in the measured time and thus determine that at about the 15th minute, the wear curve moved from the area of normal wear to the area of catastrophic wear. Achieving the limit wear requires stopping the processing process. In order to illustrate this, there were two states at the 15th minute when the process should have stopped (Figure 8). For research reasons, the cutting process was continued to find out how the dependence between sound and wear develops in an extreme situation. Based on the obtained measurement results, it was then possible to search for the connections in question, especially between sound and wear, and then examine the connection of the created surface quality.

The correlation of the generated sound in cutting and wearing the cutting tooltip demonstrates a high degree of correlation between the change in sound development trend and the wear curve that follows from the previously presented material. Figure 8 clearly shows that at the 15th minute, the wear curve moved from the area of normal wear to the area of catastrophic wear, which requires stopping the metalworking process. This method of early diagnosis of the tool wear condition using the *a_qp_* parameter is very important for the retrograde regulation of the generated surface quality during the timely measurement of sound. Thus, it is possible to achieve the highest-quality process emphasising the quality of the created surface represented by the surface roughness.

### 4.2. Experiment with the Cutting Insert T15K6

**Figure 9:**
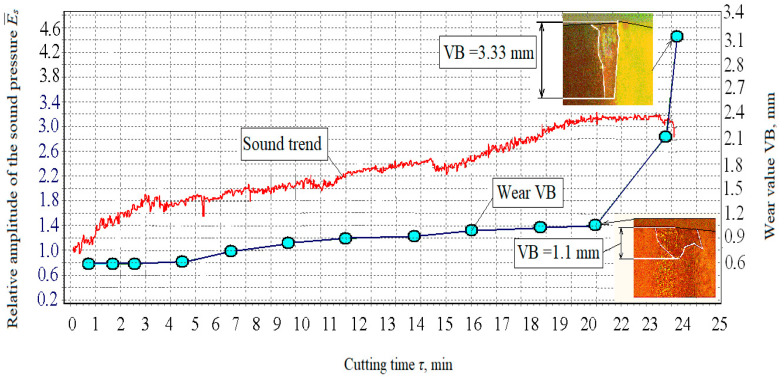
Sound trend and wear curve.

The size trend and the wear curve of the cutting insert (flank width VB) are shown in Figure 9. Here, the link between sound and wear by another blade was identified. In order to search for and examine the relationships between the parameters in question, a symmetrical image of the sound trend (Figure 9) is presented by a curve that changed at the same distance from the concerned significant change in the wear curve portion corresponding to the usual wear portion. The consistency of the change in the sound trend and the wear curve was clearly confirmed (Figure 9) by the relationship between the amplitude of the sound wave and the wear rate.

**Figure 10:**
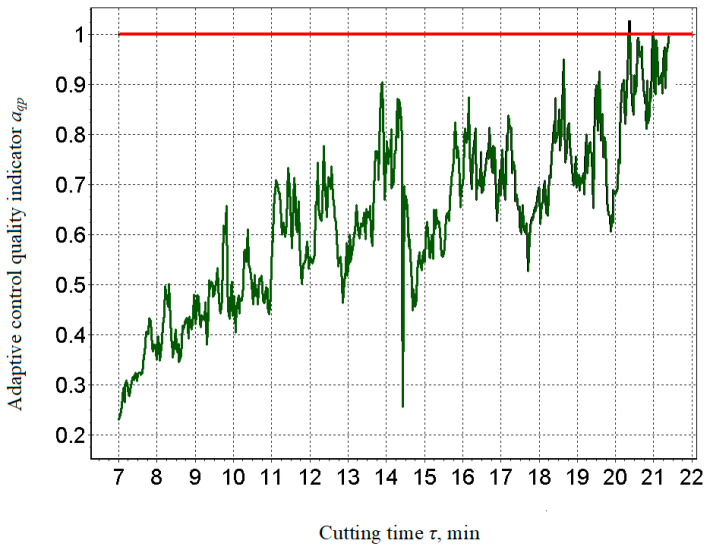
Quality indicator for adaptive control *a_qp_*.

The analysis shows that at the 20th minute, the wear curve shifted from the area of normal wear to the area of catastrophic wear, which requires the metalworking process to finish, and this was also evidenced by the change in machining quality index *a_qp_*, which reached a critical value equal to one (Figure 10). The moment it corresponded to the transition from the faultless operation of the processing system to the defective state was specifically evident in the trend at 20.2nd minute when a failure occurred, i.e., at the time of the tool failure, the machining quality index aqp exceeded the maximum allowable level. In this way, it is possible to predict when the process needs to be stopped and the blade replaced, which adequately corresponds to relations (10) and (11).

### 4.3. Correlation between Sound and Roughness

In order to compare the roughness *Ra* and the corresponding acoustic parameter $\bar{R}a$ in a dimensionless form, the definition relation (14) is used:

(14) $$\bar{R}a=\frac{Ra\left(\tau \right)}{Ra\left(\tau_{0}\right)},$$

where *Ra* (*τ*_0_) is the height roughness parameter defined at the beginning of the cutting process, and *Ra* (*τ*) is the height roughness parameter defined at the current cutting process time.

The experimental results also made it possible to define the relation (15) characterising the coordinated evolution of the sound wave amplitude and height roughness parameter during processing:

(15) $$\frac{Ra(\tau )}{Ra(\tau_{0})}\approx \frac{E_{Si}(\tau )}{E_{S0}(\tau_{0})},$$

If the fraction on the left in relation (15) can be considered an analytical expression for the required “member function”, the relation for calculating the numerical value of the roughness quality index *a_R_ (τ)* (16) can be determined:

(16) $$a_{R}(\tau )=\frac{E_{3B}(\tau )}{E_{3B}(\tau_{0})}.$$

If the ratio (16) is solved relative to the current value of the height parameter, a roughness quality index with a length dimension of the order of μm (17) can be obtained:

(17) $$a_{R}(\tau )=Ra(\tau_{0})\cdot \frac{E_{Si}(\tau )}{E_{S0}(\tau_{0})}=Ra(\tau_{0})\cdot {\bar{E}}_{S}(\tau ).$$

In controlling the dynamic behaviour of the processing system interactively, the roughness quality index is easier to evaluate by the relation (17) because it maintains the roughness dimension. Conversely, in automatic control, it is appropriate to select a dimensionless form of its representation (16), especially with respect to the corresponding software product that is part of the software environment of a specific automatic control system for the dynamic behaviour of the processing system.

**Figure 11:**
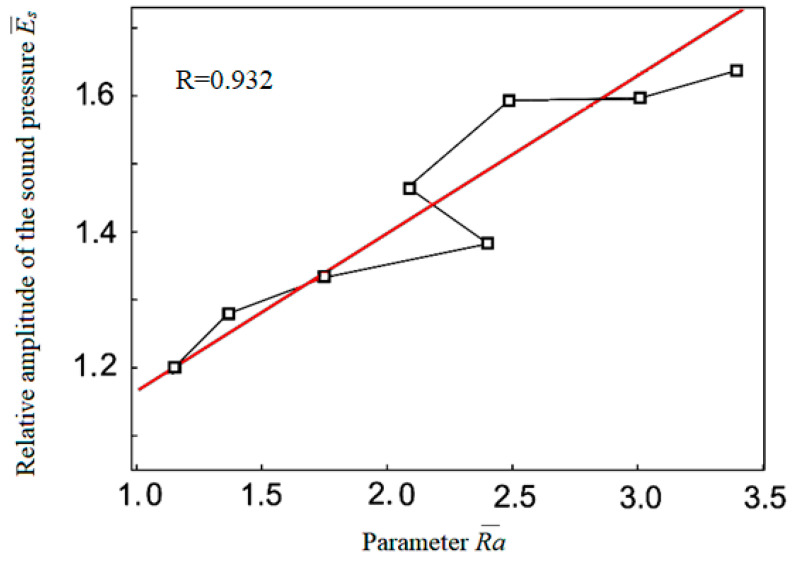
Roughness $\bar{R}a$ and sound $\bar{E}s$ (parameters) dependence.

**Figure 12:**
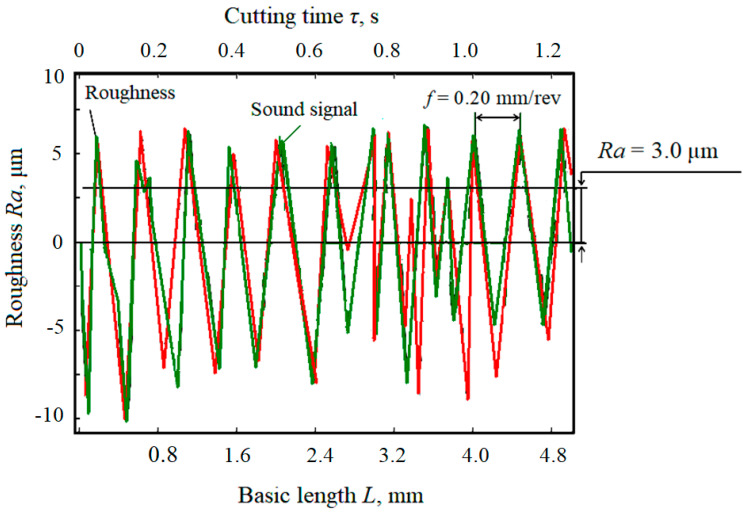
Course of roughness and sound signal depending on basic length *L* and cutting time *τ*.

Thus, the results of the experiments show a close correlation of the generated sound during cutting with the tooltip wear, as well as the correlation with machined surface quality (Figure 11 and Figure 12), clearly demonstrating the efficiency of using the cutting sound as an information signal in the process of turning. It clearly shows the efficiency of using cutting sound as an information signal in turning materials.

Figure 12 compares time and length parallel changes in the sound signal and the machined surface roughness. During the machining process, the machining tool moves in deformation contact with the material being machined, so a very intensive friction process takes place. Acoustic changes occur due to friction. These changes propagate both through the material and beyond the material through the material environment, i.e., air. The three-dimensional successive mechanical longitudinal sound wave propagates through space, while for its quantitative description, a relatively simple and precisely measurable time change of the air pressure is selected in technical practice. In the case of the machining process, this change is adequate to the cause, i.e., the change in the surface roughness. For the quantitative description of changes in the surface texture, a relatively simple and precisely measurable time change in surface roughness was chosen in technical practice. Figure 12, as obtained from experimental results, is also correlated with theoretical, analytical relations (15) to (17). The parameters of sound pressure and surface roughness change simultaneously with the change of the measured length of the sample of the machined material. The time delay between the two signals is negligible in fractions of a second of the measured time and in fractions of a millimetre of the measured length.

It can be clearly stated that a smooth surface correlates with low wear of the tip of the instrument used and at the same time with a weaker intensity of sound, i.e., with its smaller amplitude. On the contrary, the roughened surface correlates with higher wear of the tip of the used tool; the blunt tool crushes the surface. This fact naturally manifests itself in the increased intensity and amplitude of the sound. Figure 12 thus declares a clear link between the sound and surface roughness, which can be used for retrograde control and regulation of the machining process, i.e., in terms of adaptive control for a timely cutting-edge replacement.

The uncertainty of the measurement results is based on the type B uncertainty, i.e., the accuracy of the instruments used. The CNC machine operates with an accuracy of 0.001 mm, which indicates machining accuracy with a tolerance of less than one micrometre. The electret microphone achieves a frequency response ripple of ±0.5 dB in the band 5 to 30 kHz, and the sensitivity at an acoustic frequency of 1 kHz of an electrical voltage of 1 to 10 mV per acoustic pressure was 1 Pa. Surface roughness measurements were made with a profilometer measuring the vertical difference between the high and low points of the surface in nanometres.

## 5. Discussion

This article discusses an adaptive cutting control system that uses cutting sound as an interactive feedback signal for the first time. The sound is recorded in a non-contact manner, which makes it possible to eliminate disturbing effects on the recorded signal (e.g., due to machine vibrations) and thus to eliminate cutting process control errors promptly. For this purpose, a computer program has been developed to complement the standard CNC machine tool software.

### 5.1. Comparison of Research Results with Other Authors

The presented results were compared and verified [61,62], and a positive correlation between sound level, tool wear, and surface roughness of the component was clearly demonstrated. The work [61] showed that as the cutting parameters change, the sound pressure levels of the cutting process also change adequately. A comparison of the graphs in Figure 9 [61] and Figure 10 shows the identity of the information provided and indicates a close correlation between sound trends and cutter wear. This information served as the basis for the use of sound as an information signal in the development of a system for adaptive control of the cutting process.

The results [62] show that the emitted sound could be used to monitor the wear status of the tool flank during the turning process. It can be concluded that, under the circumstances, just monitoring the state of tool flank wear generated by sound is a feasible and relatively simple method. Another advantage of this method is that the time required to decide on the condition of the tool is very short, in the order of 1 s. Therefore, subjecting the signal part of a second to the presented method is a convenient and highly reliable process.

Based on the obtained results and comparison with other authors [61,62] (especially [62]), it can be concluded that in numerical control, the machining strategy is focused only on achieving the desired precise geometric shape, but in adaptive control, it is additionally focused on controlling the technological parameters of the machining process. The sound waves generated as noise due to mechanical vibrations of the machine are a verified information source. The method of non-contact sound measurement by means of a microphone placed close to the cutting area and the definition of a dimensionless comparative indicator can provide accurate results of a multiplicative and additive nature, namely noise-proof results. It has been clearly demonstrated that the trends of changes in the sound levels (Figure 9, Figure 11 and Figure 12) generated during cutting, the roughness of the machined surface and the wear of the cutting tool are highly correlated with each other. This fact also corresponds to sound efficiency as a physical quantity ideally suited for adaptive control of the metal cutting process. The criterion for indicating the maximum allowable wear value was the step change (increase) in the cutting sound in the ongoing machining time.

While the sound pressure levels generated by the cutting process change accordingly with the change of the cutting parameters, from a physical point of view, the cutting sound is only generated due to the intense friction between the workpiece and the tool, i.e., it is generated depending on the interaction of the currently acting cutting parameters. This specific friction generates heat [65,66], the main sources of heat being in the area of plastic deformation during chip formation, in the area of chip friction on the tool face, and in the area of back friction on the machined surface. The level of sound pressure generated during metalworking is highly variable and can, for example, reach a maximum of 80–85 dB. In the case of the presented method, however, it is not about quantitative values of the measured acoustic parameters but about their qualitative changes. The point is that sound trends correlate with trends in roughness and wear. 

### 5.2. Application of the Obtained Results

In the future, it can be expected that there will be a sufficient number of applications in the practice of metal cutting and that the development of the investigated problem will move to the level of optimisation adaptive control. In particular, it will be necessary for adaptive optimisation systems to obtain information about the extent of tool wear and the tool wear rate.

The new research approach of adaptive control will necessarily be part of the research on automation and robotisation of the cutting process of CNC machines. The comparison of cutting sounds generated during machining of different materials has not been carried out but will be the subject of further research.

The possibilities of practical applications of the implementation of the proposed method are considerable. Current machine tools are equipped with G code (ISO code), i.e., a programming language used to execute motion instructions and other auxiliary instructions for the machine tool. The basic principle of G-code programming is common to both lathes and milling machines (the programming of turning and milling operations differs at the level of specific instructions for each operation).

The application of the method is limited by the type of machining that has been studied so far, i.e., chip-forming machining. For example, in ultrasonic machining, tool wear is also dependent on the workpiece material, tool material, and working conditions, but an ultrasonic microphone would need to be used [67].

The use of the cutting tool as a sound source and the adaptive control of the machining process in terms of the declared correlations consists of approximating the adopted trend model of the results of the sound pressure monitoring accompanying the cutting process by means of a graph. The quality of the approximation is evaluated by the degree of correlation between the calculated and measured trends in the amplitude of the sound wave. In addition, the correlation between the calculated and measured data in the area of the trend behaviour is crucial in the prediction. An acceptable level of correlation in a given area, in turn, determines the quality (reliability, accuracy) of the source prediction. It is, therefore, a predictive and diagnostic–experimental complex, which enables real-time technical condition monitoring and adaptive control of processing systems without interrupting the processing of materials by cutting. The predictive and diagnostic complex makes it possible to realise one of the important tasks of modern production, automation, in engineering practice. The predictive–diagnostic complex of the machining process will enable:

- The expansion of the functionality and increasing the productivity of processing systems;
- Improvement in the quality of operation of processing systems and elimination of their unplanned shutdown in order to replace defective elements;
- The improvement in working conditions, increasing work safety and environmental friendliness of processing systems through the operational management of their work processes, which is the main goal of the modern metalworking industry.

The purpose of the work was not to create a regression equation that allows one to determine the value of the roughness and the amount of tool wear by the magnitude of the sound pressure. The work aimed to develop a technique for controlling the cutting process. This technique makes it possible to predict the timely replacement of a worn tool.

## 6. Conclusions

The article presents a new method of effective use of the acoustic signal generated during the cutting process, particularly to create the desired surface properties of the workpiece. The research aimed to find and verify the correlation relationship between the sound generated during the turning process, tooltip wear, and workpiece surface roughness.

The article clearly discusses that as the cutting parameters change, the sound pressure levels of the cutting process also change adequately; if the cutting process is not in progress, the overall sound pressure level is minimal; if an adverse event occurs in the cutting process, the sound pressure level suddenly increases or decreases. Therefore, an available method for developing an alarming system is reducing the sound pressure level.

The results of experimental studies were realized on the workpiece material of 12X18H10T steel using two cutting inserts P25 and T15K6. The correlation of sound trends and trends of surface changes of the machined material, i.e., its roughness and wear, were predicted, verified, and confirmed. Confirmed compliance offers the advantageous use of the audio signal as a starting point for adaptive control of materials machined by cutting with the following conclusions:

- Unlike the data signals obtained by the contact method and thus subject to noise generated by the mechanical vibration of the machine, the non-contact sound measurement method using a microphone located close to the cutting area and the definitions of a comparative dimensionless indicator of the accuracy of measurement results processing provide noise-resistant results of multiplicative and additive character, as well as results resistant to pulse disturbance.
- The development trend of the generated sound and the trend of the tooltip wear change during the cutting process and show a high degree of correlation, as evidenced by the high value of the correlation coefficient (R = 0.93).
- Moreover, a strong correlation was found between the sound trend and surface roughness parameter *Ra* characterised by a relatively similar correlation coefficient (R = 0.93).

We have developed a technique for controlling the cutting process using the sound that is generated during the metalworking process. Based on the results, the accuracy of the proposed method can be declared by the user up to 5%. Therefore, in this way, the instantaneous values of the measurements represent a sufficiently accurate actual state and connections of individual parameters. It is a fundamental difference from current methods of adaptive control of the cutting process, which use the mean values and regression equations, but they change when the technological conditions change. It is a disadvantage of current methods of adaptive control.

In conclusion, the data presented in this paper justify the recommendation of a non-contact method of measuring the sound generated during turning for the process of adaptive control of material processing.

## Figures and Tables

**Table 1:** Experimental conditions implemented during turning.

Cutting Inserts	Cutting Conditions			*D*, mm
	*n*, rpm	*f*, mm/rev	*a_p_*, mm	
P 25	125	0.15	1	98.0
T15K6	315	0.20	1	

## Data Availability

The data that support the findings of this study are available from the corresponding author (VN) upon reasonable request.

## Nomenclature

**Symbol**		**Measurement Unit**
*a_p_*	cutting depth	mm
*f*	feed rate	mm/rev
$E_{S}$	sound wave pressure signal	Pa
$E_{Si}$	the measured value of the sound signal	Pa
$E_{S0}$	the measured initial value of the sound signal	Pa
${\bar{E}}_{S}$	relative sound signal	-
${\bar{E}}_{S}(\tau )$	the relative value of the sound pressure signal relative to time *τ*	-
$E_{Si}(\tau )$	the measured value of the sound signal relative to time τ	Pa
$E_{S0}(\tau_{0})$	the measured initial value of the sound signal relative to time *τ_0_*	Pa
${\hat{E}}_{S}$	the root-mean-square value of useful signal and interference	Pa
${\hat{E}}_{Si}$	the root-mean-square value of the measured value of the sound signal	Pa
${\hat{E}}_{S0}$	the root-mean-square value of the measured initial value of the sound signal	Pa
${\hat{E}}_{S}^{SUM}$	the total effective sound pressure value at the control point	Pa
$E_{3B}$	the magnitude of the amplitude of the sound wave	Pa
$E_{3B}(\tau )$	the magnitude of the amplitude of the sound wave relative to time *τ*	Pa
$E_{3B}(\tau_{0})$	the magnitude of the amplitude of the sound wave relative to time *τ_0_*	Pa
$E_{Sh}$	real-time audio signal of the cutting process response in real time	Pa
$n_{PCS}$	number of read signal values	-
ε(τ)	the duration of the multiplicative noise	min
$\hat{\varepsilon }(\tau )$	the root-mean-square value of the duration of the multiplicative noise	min
τ	the time of the current measurement to which the quantities relate	min
$\tau_{0}$	the time of the initial measurement to which the quantities relate	min
$\tau_{Sh}$	the *E_Sh_* pulse duration	10^−4^ s
Δτ	PC processing time step	10^−5^ s
T	average predicted trouble-free operation time of machining systems using cemented carbide cutting tools from the current batch of samples	min
${\bar{T_{}}}_{\exp}$	average experimental time of failure-free operation/ numerical value of tool life	min
L	length of the bar (in terms of dynamic characteristics, equivalent to a metal object that produces a sound impulse when dropped on the floor, for example)	m
C	speed of propagation of longitudinal vibrations in metal	m∙s^−1^
$a_{qp}$	machining quality index, an indicator for adaptive control	-
$h_{r}$	the amount of dimensional wear in the blade tip area	mm
$h_{r}(\tau )$	the amount of dimensional wear in the blade tip area relative to time *τ*	mm
$h_{r}(\tau_{0})$	the amount of dimensional wear in the blade tip area relative to time *τ**_0_*	mm
δ	the degree of change in the geometry of the workpiece component due to tooltip wear	-
$D_{\Phi }$	the actual size of the workpiece component	mm
$D_{H}$	the nominal size of the workpiece component	mm
$D_{H}^{+\Delta B}$	the nominal size of the workpiece component relative to the Δ*B-value*	mm
ΔB	change in radial tool wear	mm
$\bar{R}a$	surface roughness of the workpiece	μm
$\bar{R}a(\tau )$	workpiece surface roughness relative to time *τ*	μm
$\bar{R}a(\tau_{0})$	workpiece surface roughness relative to time *τ**_0_*	μm
$a_{R}(\tau )$	the numerical value of the roughness quality index	-
